# Telomere length in granulosa cells and leukocytes: a potential marker of female fertility? A systematic review of the literature

**DOI:** 10.1186/s13048-020-00702-y

**Published:** 2020-08-21

**Authors:** Anne-Julie Fattet, Simon Toupance, Simon N. Thornton, Nicolas Monnin, Jean-Louis Guéant, Athanase Benetos, Isabelle Koscinski

**Affiliations:** 1 Laboratory of Biology of Reproduction-CECOS Lorraine, University Hospital of Nancy, 10 rue du Dr Heydenreich, 54000 Nancy, France; 2 Université de Lorraine, Inserm, DCAC, F-54000 Nancy, France; 3 Centre AMP Majorelle-Laboratory ATOUTBIO, 95, rue Ambroise Paré, 54000 Nancy, France; 4 Université de Lorraine, Inserm, NGERE, F-54000 Nancy, France

**Keywords:** Telomere length, Telomerase, Premature ovarian failure, Primary ovarian insufficiency, Premature ovarian insufficiency

## Abstract

In the context of a continuously increased delay of motherhood and of an increase of the incidence of premature ovarian failure, it is of the greatest interest to dispose of a predictive marker of the duration of the fertility window. Unfortunately, current available markers of women’s fertility (hormonal rates or echography count of small follicles) have a poor predictive value of premature ovarian failure. In the last ten years, some studies have suggested that telomere length may be correlated with premature ovarian failure, but the results of these studies are contradictory.

In accordance with guidelines from Preferred Reporting Items for Systematic Reviews and Meta-Analyses (PRISMA), this systematic review of the literature selected studies evaluating telomere length or telomerase activity in granulosa cells and/or in leukocytes as a premature ovarian failure marker.

Five publications (252 premature ovarian failure patients) were included in this review of experimental evidence. Two of them studied telomere length and/or telomerase activity in granulosa cells and 4 in leukocytes in women with premature ovarian failure. For each study, authors determined if there was a positive or a negative correlation between telomeric parameters and premature ovarian failure.

3 studies (178 premature ovarian failure patients) found shorter telomere length in granulosa cells and/or leukocytes and/or lower telomerase activity in premature ovarian failure patients. 2 studies (74 premature ovarian failure patients) presented contradictory results about the correlation of leucocyte telomere length with premature ovarian failure.

Shorter telomeres and diminished telomerase activity in granulosa cells appear to be associated with ovarian insufficiency. However, the number of studies and of subjects within are low and the methodology questionable. The confirmation of these results is essential with more subjects, better defined populations and more adapted methodology, in order to consider telomere length in granulosa cells and/or in leucocytes as an early and reliable marker for the decline of ovarian function.

## Background

Over the past thirty years, in most developed countries, women are having children later and later. But, at the same time, they are also becoming more aware that their fertility declines with age. This could be the reason why many of them would be in favour of fertility preservation to ensure that they will be able, one day, to become mothers [1].

Premature Ovarian Failure (POF), first described in the 1930s, is a clinical syndrome characterized by a loss of ovarian function before the age of 40 [2] with three sequential stages called occult, biochemical and clinical [3]; corresponding to fertility decline, then an increase in Follicle Stimulating Hormone (FSH) release rate (twice), and finally oligo or amenorrhea [4] respectively. The prevalence of POF among women under 40 years of age is about 1%. Furthermore, it is estimated at 1/1000 for those under 30 years and 1/10,000 for those under 20 [5, 6]. About 10 to 28% of women with primary amenorrhea have POF; and in women with secondary amenorrhea, the frequency is between 4 and 18%. However, prevalence varies depending on population characteristics with such factors as ethnicity. Indeed, the frequency is higher among Caucasian and African than Asian women [6]. Although the definition of POF is incomplete, the European Society of Human Reproduction and Embryology (ESHRE) working group recommends as diagnostic criteria [4]: Oligo/amenorrhea for at least 4 months and FSH > 25 IU/L at twice 4 months apart.

Climacteric symptoms are less common in patients with primary amenorrhea, suggesting that they are mainly due to a cessation of estrogen excretion rather than estrogen deficiency [6].

Long-term consequences of POF are summarized in Table 1 [7]: Estrogen deficiency leads to a reduction in bone mineral density and therefore to an increased risk of osteopenia, osteoporosis and fracture with age. These patients have also an increased risk of developing cardiovascular diseases [8] and an increased risk of all-cause mortality. This pathology has also an undeniable negative impact on the psychological well-being of patients [6, 9]. In a large majority of cases the aetiology of this pathology remains unexplained [10]. Nevertheless, in some patients with POF, genetic abnormalities [7, 11–13], metabolic disorders, autoimmunity, iatrogenic, infections or environmental factors have been defined as underlying causes of this syndrome [6].

**Table 1:**
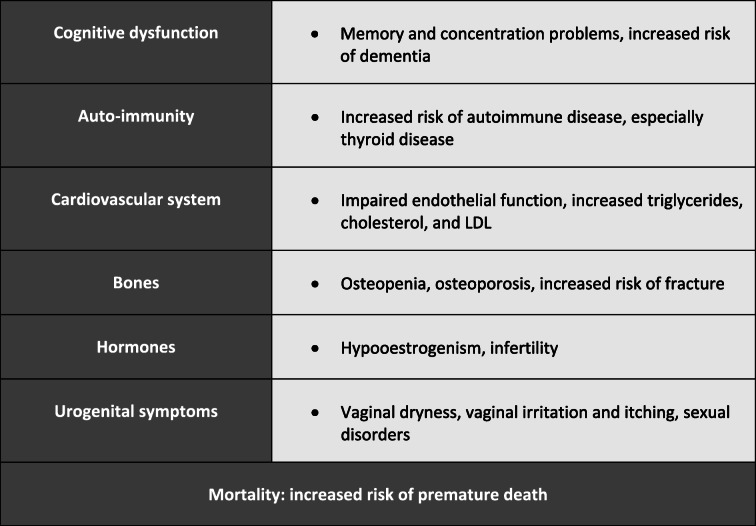
Summary of long-term consequences of premature ovarian failure, according to [7]

According to different authors, the discriminative value for blood FSH varies from 25 to 40 IU/L.

After the diagnosis the following dosages are typically performed [14]: Anti-müllerian hormone (AMH): low; Luteinizing Hormone (LH): increased and estradiol: low, especially with a view to a treatment [15].

Currently, there is no treatment to improve ovarian function or spontaneous pregnancy rates in women with POF (between 5 and 10% in idiopathic POF) [15] and no test or marker that predicts the risk of developing POF [4], except for the search for a few mutations known to cause this disease. As POF markers, AMH levels and antral follicle count (AFC) are partially predictive of the outcome of Assisted Reproductive Technology (ART) in patients consulting for infertility, but no predictive markers of the risk of developing a POF several years later. The only preventive measure that can be proposed to future POF patients is female fertility preservation [4] either with ovarian cortex or with oocytes and embryo cryopreservation. In addition to the cost, these different techniques of fertility preservation are not without risks to health. Therefore, it would be particularly interesting to find a predictive marker for the development of POF.

The term “telomere” derives from the Greek words “*telos*” which means “end”, and “*meros*” which means “segment”. Telomeres were first identified 80 years ago by Hermann Muller working with the fruit fly (*Drosophila melanogaster*) and by Barbara McClintock working with corn (*Zea mays*) cells. Telomeres are specialized non-coding double-stranded repetitive DNA-protein complexes that form protective caps at the ends of eukaryotic chromosomes [16]. These heterochromatic structures are composed of guanine rich TTAGGG repeats associated with a family of proteins known as the shelterin complex (TRF1, TRF2, POT1, TIN2, TPP1, and Rap1) [17]. These structures maintain genomic integrity through their capacity to prevent their recognition as a DNA double-stranded break and thence activation of DNA damage response (DDR), or end-to-end fusions [16]. During each cell division, part of the DNA located at the chromosomes’ extremity is lost due to incomplete replication of the lagging strand, a phenomenon known as the “end replication problem” [18]. In somatic cells, this leads to progressive telomere attrition/shortening and in the end to critically short telomeres, which triggers replicative senescence or apoptosis [19].

Telomerase is a ribonucleoprotein composed of a catalytic subunit called “Telomerase Reverse Transcriptase” (TERT) and an RNA matrix subunit called “Telomerase RNA component” (TERC). This RNA-dependant DNA polymerase can maintain telomere length by extending the guanine-rich single strand of telomeres, allowing DNA polymerase to achieve synthesis of the opposite strand and thus avoids the progressive loss of DNA at each replication cycle [20]. Telomerase is repressed in somatic cells but active in embryonic, stem and germ cells, as well as in 90% of tumor cells [21]. This activity prevents telomere shortening with cell division, preventing cell senescence and thus allowing extensive proliferative capacities [16].

In humans, mean telomere length ranges from 4 to 12 kb in somatic cells and from 10 to 20 kb in germinal cells [22, 23]. These mean values differ within an individual depending on the cell type, tissues, and organs. Leukocyte telomere length (LTL), the most studied telomere length in clinical and epidemiological studies due to the easy accessibility of leukocytes, decreases with age [24]. The LTL attrition rate is higher in utero and during the first years of life and then it decreases during adulthood [25–27]. In adults, LTL attrition rates are estimated at 25 to 35 bp/year [28]. LTL is highly heritable (60 to 70%) and displays a wide range of mean values (4 kb range) between individuals of the same age in a population [22, 23, 29]. This variation is essentially determined by genetic factors through different genes, located on both autosomal and X chromosomes. However, other factors such as increased paternal age, female sex and African ancestry also contribute to TL [16].

LTL is typically shorter in men than in women, the mean difference being approximatively 200 bp [30]. The origin of this gender gap is under debate but one of the hypotheses is that it appears at puberty due to the estrogen rise in women. Women’s telomeres could be protected by the antioxidant properties of estrogens through stimulating manganese superoxide dismutase and glutathione peroxidase via Mitogen-Activated Protein kinases (MAP-kinase) and Nuclear Factor- κB (NF-κB) pathways [31]. Moreover, estrogens could also directly activate telomerase via the *hTERT* promoter given that an estrogen-response element is present in this promoter [32]. Therefore, studying the physiological aging process through the telomere spectrum opens up a new approach to female fertility exploration [33].

### Rationale of systematic review of the literature

A recent study on telomere length in germ cells suggested that this parameter could be a biomarker of germ cell and embryo quality [34]. Telomeres play also a role in fertility and there might exist a positive or negative association between relative TL and different factors of female and male infertility [35].

Indeed, germ cell mitotic activity is very different according to gender: male stem germ cells are constantly dividing, and the stock stays constant during the lifespan from puberty onwards. On the other hand, female germ cells stop their division before birth. On the contrary, granulosa cells must divide slowly but regularly to maintain a stock of granulosa cells around the oocytes in primordial and primary follicles from the 7th month of intra-uterine life until the menopause, and intensely during follicular maturation [34, 36]. Therefore, in the context of female fertility, it would appear to be more interesting to investigate telomere length in granulosa cells than in germ cells. Short telomeres could impact female fertility by limiting mitosis ability of granulosa cells twice in a woman’s life:

- Continually during a woman life, when granulosa cells multiply in order to maintain a pool of follicles
- Each month, when granulosa cells multiply under gonadotropins stimulation in order to develop a follicle until dominance and ovulation

Several human studies have tried to highlight a correlation between telomere length and female infertility. The following literature review evaluates the potential link between telomere length and/or telomerase activity in granulosa cells and leukocytes in women with POF.

## Material and methods

**Fig. 1:**
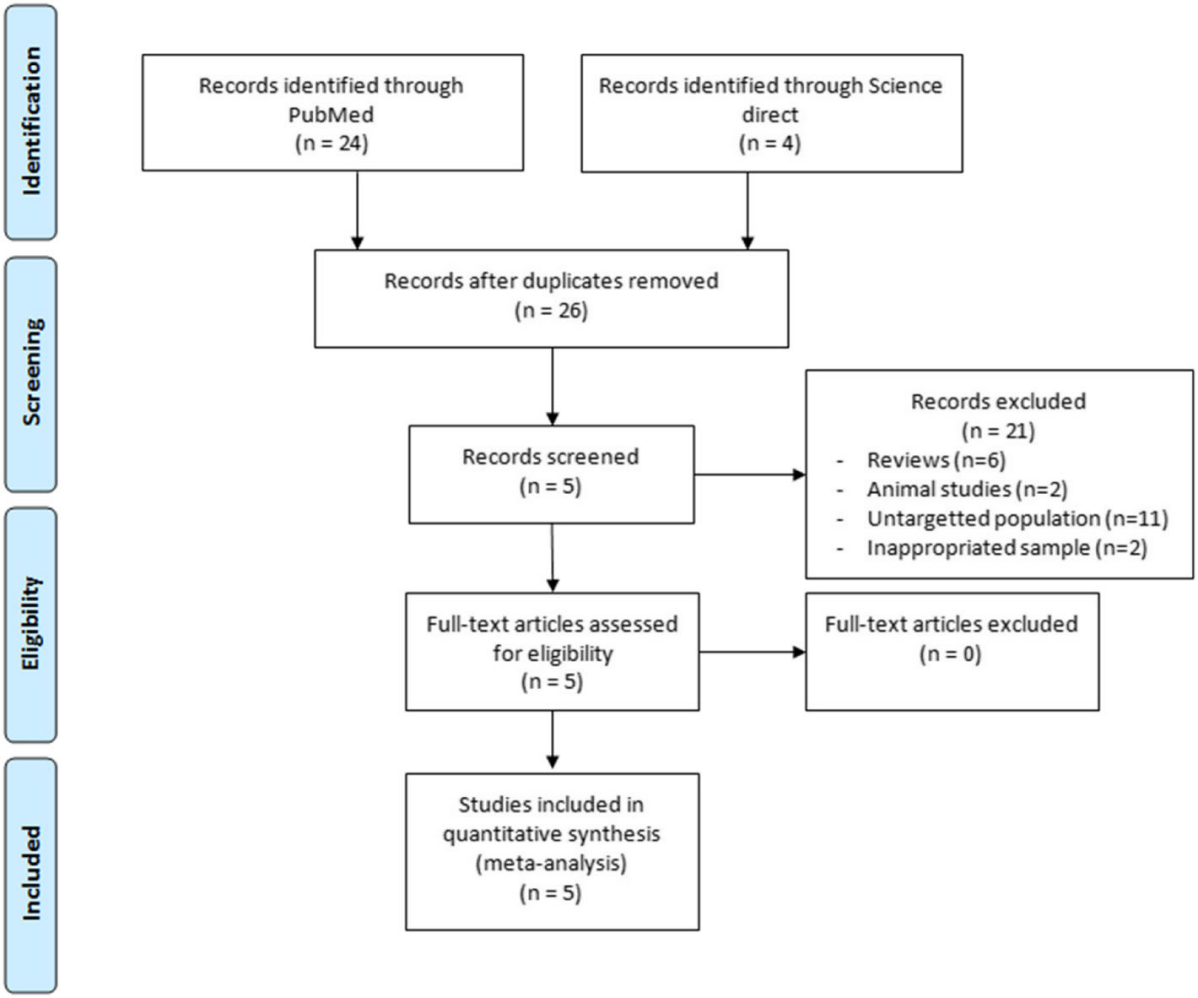
PRISMA Flow diagram for identification and selection of studies

A systematic review of the published literature relating to the link between telomere length and female fertility has been performed following the PRISMA guidelines (« Preferred Reporting Items for Systematic Reviews and Meta-analyses ») [37] (Fig. 1) using online search of PubMed and ScienceDirect at the date of 01/12/2019 to identify all experimental studies. The most exhaustive research was made by using the keywords: (telomere length OR telomerase) AND (primary ovarian insufficiency OR premature ovarian failure OR occult ovarian insufficiency OR hypergonadotropic ovarian failure OR premature ovarian insufficiency).

We identified 28 hits and screened the titles and/or abstracts to assess eligibility. A total of 5 publications were included in the review of experimental evidence. Two of them studied telomere length and/or telomerase activity in granulosa cells and four of them in leukocytes in women with POF.

For each study, authors determined if there was a positive or a negative correlation between telomeric parameters and premature ovarian failure.

Data on publication date, study objective, sample, selection criteria, number of subjects, age, methods and analysis, study outcome were extracted and resumed in the tables [38–42].

## Results

### Subjects

Characteristics of the 796 participants in the 5 studies and criteria dividing them into POF and control groups are presented in Table 2.

**Table 2:** Characteristics of patients with biochemical POI and controls in the different studies

Studies	Number	Criteria of patient’s inclusion	Age
Butts et al. 2009 [38]	12 POF	FSH ≥ 11,4 IU/L and estradiol ≤293,6 pmol/L	30–37
	42 controls	Normal FSH, tubal factor or male infertility	23–37
Hanna et al. 2009 [39]	34 POF	Three-month amenorrhea, FSH > 40 IU/L before the age of 40	21–50
	108 controls	Overall population	17–55
Xu et al. 2017 [42]	120 POF	Regular cycles, FSH ≥ 10 UI/L, unilateral AFC < 5	≤ 40
	279 control (leukocytes) 90 controls (granulosa)	Lack of criteria	
Miranda-Furtado et al. 2018 [40]	46 POF	Amenorrhea, FSH > 40 UI/L before the age of 40	18–41
	75 controls	Regular cycles, FSH < 10 UI/L	
Sayban et al. 2018 [41]	40 POF	Six-month amenorrhea, FSH > 40 UI/L before the age of 40	< 40
	40 controls	Regular cycle, normal FSH	

### Telomere length

In the 5 studies, telomere length was assessed using a modification of the quantitative PCR method described by Cawthon [43]. After extraction, telomeric DNA was amplified by PCR in the same time as a single copy gene. This technique estimates telomere length by comparing the amount of telomere repeat amplification product (T) to the amount of the single copy gene (S) product. The Telomere/Single Copy Gene ratio (T/S ratio) correlates with the average telomere length [38–42]. In the two studies measuring TL in granulosa cells, TL was shorter in POF patients than in controls (Table 3). The 4 studies measuring LTL reported conflicting findings. In 2 of them LTL was shorter in POF patients than in controls whereas the 2 other studies reported longer LTL in POF patients.

**Table 3:** Results for telomere length

Studies	Samples	Results
Butts et al. 2009 [38]	Granulosa cells	POF: 1,88 ± 0,69^a^
		Controls: 3,15 ± 0,25
Hanna et al. 2009 [39]	Leukocytes	POF: 9,61 ± 1,38^b^
		Controls: 8,98 ± 1,15
Xu et al. 2017 [42]	Granulosa cells	POF: 0,78 ± 0,09^c^
		Controls: 1,90 ± 0,23
	Leukocytes	POF: 0,75 ± 0,09^d^
		Controls: 1,79 ± 0,12
Miranda-Furtado et al. 2018 [40]	Leukocytes	POF: 0,93 ± 0,23^e^
		Controls: 1,07 ± 0,27
Sayban et al. 2018 [41]	Leukocytes	IOP: 0,7445^f^
		Controls: 0,5994

^a^*p* = 0,039

^b^*p* = 0,01

^c^*p* < 0,001

^d^*p* < 0,001

^e^*p* < 0,0006

^f^*p* < 0,05

### Telomerase activity

Telomerase activity was measured in granulosa cells in a subgroup of 123 participants in two studies (Table 2). Telomerase activity (TA) was detected using a modified telomeric repeat amplification protocol (TRAP). Presence or absence of TA was determined based on the characteristic laddering pattern of telomerase products [38] or relative TA quantified based on a standard curve [42]. In both studies, TA activity was higher in granulosa cells from controls than in those of POF patients (Table 4).

**Table 4:** Results for telomerase activity

Authors	Expression	Results	
Butts et al. 2009 [38]	Absence of telomerase products	POF: 11/12 (92%)	OR (95%): 11^a^
		Controls: 21/42 (50%)	
Xu et al. 2017 [42]	Relative telomerase activity	POF: 1,57 ± 0,59^b^	
		Controls: 4,63 ± 0,93	

^a^*p* = 0,02

^b^*p* = 0,025

Table 5 presents the overview of the statistical results obtained in the five studies concerning TL and TA in granulosa cells and leukocytes in both POF and control groups.

**Table 5:** Summary table of the different studies

Authors	Samples	Subjects	Setting	Method	Results
Butts et al. 2009 [38]	Granulosa cells	12 POF 42 controls	TL	qPCR	Shorter telomere in POF
	Granulosa cells	12 POF 42 controls	TA	PCR TRAPeze Telomerase Detection Kit	Absence of TA more frequent in POF
Hanna et al. 2009 [39]	Leukocytes	34 POF 108 controls	TL	qPCR	Longer telomere in POF
Xu et al. 2017 [42]	Granulosa cells	120 POF 90 controls	TL	qPCR	Shorter telomere in POF
	Leukocytes	120 POF 279 controls	TL	qPCR	Shorter telomere in POF
	Granulosa cells	31 POF 38 controls	TA	PCR Q-TRAP	TA level lower in POF
Miranda-Furtado et al. 2018 [40]	Leukocytes	46 POF 75 controls	TL	qPCR	Shorter telomere in POF
Sayban et al. 2018 [41]	Leukocytes	40 POF 40 controls	TL	qPCR	Longer telomere in POF

*TL* Telomere length

*TA* Telomerase activity

## Discussion

The studies of Butts et al. and of Xu et al. demonstrated that telomeres are shorter in granulosa cells (GC) of women with POF than in those of healthy controls. Moreover, these two studies observed also decreased telomerase activity in GC from POF patients. These results suggest that short telomeres in GC are associated with a fertility decrease. Therefore, telomere length in GC should be considered as a new marker of reproductive lifespan in women [38, 42].

The hypothesis to explain the impact of short telomeres in GC on female fertility could be through the limited proliferation capacity of cells with short TL [44]. The limited proliferation capacity of granulosa cells has been demonstrated to be a characteristic that differentiates women with POF from fertile women. The development of ovarian follicles requires an acute increase of granulosa cell number from a few cells to tens of thousands before ovulation [38]. Moreover, an insufficient activation of telomerase in early follicular development stages could accelerate telomeric attrition and severely compromise cellular functions necessary for good follicular maturation [38, 45, 46]. For many years, granulosa has been considered as containing cells with multipotent stem cell characteristics [47], as the possibility of dividing without the need of anchorage [48] and differentiation into varied cell types [49]. As other stem cells, these cells present a constitutionally high telomerase activity [50, 51]. The pattern of this telomerase activity has been studied through the different types of follicle in ovaries and with aging, especially in bovine [52]. It has been found heterogeneous: the highest levels of telomerase activity are found in the smaller rapidly growing preantral follicles [53], followed by a decrease in activity with the maturation of the follicle [45]. This suggests that the high proliferative activity of granulosa cells could be partially linked to telomerase activity [54] and supports the hypothesis that a decrease in this activity may participate in POF. However, the obtained conclusions about telomerase activity decrease in the studies reviewed are based on analyses of luteinized granulosa cells which are terminally differentiated and morphologically distinct from proliferating granulosa cells in early stage follicles [38].

The hypothesized link between LTL and female fertility is based on the premise that LTL and GTL should be correlated. It is known that all TL are synchronized in somatic tissues at birth [55] and that despite the difference in TL observed between tissues due to different proliferative indexes, strong correlations in TL across somatic tissues subsist later in life such as in individuals with long (or short) TL in one tissue also have long (or short) TL in other tissues [56]. However, this synchrony may potentially not apply to granulosa cells since TL dynamics of these cells are influenced by telomerase activity. The systematic review of the literature about LTL in women with POF presented contradictory results.

First, the studies of Hanna et al. and of Sayban et al. concluded that LTL was longer in women with POF. According to the authors, these results were not expected since they initially thought that women with POF would demonstrate accelerated cellular aging [39, 41]. Their first hypothesis to explain these results was that longer LTL reflected a decreased division rate in the early germ cell pool. As a consequence of fewer cell divisions in germ cells, the ovarian follicular pool was reduced, and patients had a higher risk of developing POF by consuming the oocyte pool [39, 41]. Secondly, the authors proposed that longer telomeres in women with POF could be the result of an auto-immune mechanism which has been found frequently in this population. This auto-immune phenomenon could change the repartition of blood cells by selecting a specific cell type with longer telomeres. Nevertheless, some studies on telomere length and auto-immune disease suggest that auto-immune diseases are associated with shorter telomeres rather than with longer ones. Thus, this explanation is unlikely [39, 41]. Finally, their third hypothesis was that long-term substitutive hormonotherapy given to these patients could slow down the telomere attrition rate by protecting telomeres from reactive oxygen species (ROS). Estrogens may increase telomerase activity by inducing hTERT expression [57]. However, the authors did not measure estrogen levels in these patients and in a study by Miranda-Furtado et al., no correlation between telomere length and estradiol levels was found. Moreover, in their study, Sayban et al. didn’t mention the mean age of participants included in POF and control groups. Since LTL is highly influenced by age, this could constitute an important bias in this study [39–41].

Unlike Hanna et al. and Sayban et al., Xu et al. and Miranda-Furtado et al., reported shorter telomeres in leukocytes of women with POF than in controls [40, 42]. These results are in line with the “synchrony hypothesis” [55]. However, these 2 pilot studies were conducted with a limited number of subjects and must be confirmed with larger number of participants especially considering the contradictory results with the 2 other studies. If these results are confirmed, this would make it possible to avoid taking granulosa cells to measure GTL. If LTL has the same association with POF as GTL, it could serve as a surrogate marker with an easier accessibility and applicability in clinical and laboratory practices. However, a recent study conducted in 35 fertile egg donors did not find any association between GTL and LTL based on qPCR measurements [58].

Interestingly, it must be noted that all of these studies were conducted with a qPCR-based method to measure TL. This method is known to have high measurement error [59, 60] and thus requires a large sample size to offset the error, which was not the case for the 5 studies reviewed nor the one comparing GTL and LTL.

Another important bias might arise from differences in inclusion criteria between the studies reviewed. For example, level of FSH mentioned as POF criteria varied from 10 IU/L in Xu et al. to 40 IU in Hanna et al., Miranda Furtado et al. and in Sayban et al. (Table 2). In Butts et al., the POF threshold was based on data from patients undergoing in vitro fertilization (IVF) with low pregnancy rate. In the Xu et al. study, the patients were presented with only biochemical criteria and not clinical symptoms of POF since they kept regular cycles. The literature does not highlight any direct link between FSH levels and telomere length. However, high FSH levels result from lower estradiol levels which could be linked to decreased telomerase activity [57] and compromised telomere protection against ROS. Moreover, long term hormone replacement could slow the kinetics of telomere attrition. Hanna et al. included patients from 21 to 50 years old and some of them certainly took hormonal supplementation. This may have introduced some bias. Finally, patients included presented different stages of POF which makes it difficult to compare these studies.

## Conclusion

As an overall conclusion of this systematic review of the literature, shorter telomeres and diminished telomerase activity in granulosa cells appear to be associated with occult ovarian insufficiency. However, the number of studies and of subjects within are very low and the methodology questionable. If these results were to be confirmed with more subjects, better defined populations and more adapted methodology, the measurement of these parameters could be an early and reliable marker for the decline of ovarian function, as suggested in a recent reflection by Pr Barlow [61]. This may also have important consequences for women’s health beyond fertility preservation. However, the question whether short telomeres observed in women with POF are a cause or a consequence of this disease remains to be addressed in future studies.

## Data Availability

Not applicable.

## Abbreviations

POF: Premature Ovarian Failure
FSH: Follicle Stimulating Hormone
ESHRE: European Society of Human Reproduction and Embryology
AMH: Anti-Müllerian Hormone
LH: Luteinizing Hormone
AFC: Antral Follicle Count
ART: Assisted Reproductive Technology
DDR: DNA Damage Response
TERT: Telomerase Reverse Transcriptase
TERC: Telomerase RNA Component
LTL: Leukocyte telomere length
TL: Telomere Length
MAP-kinase: Mitogen-Activated Protein kinases
NF-κB): Nuclear Factor- κB
hTERT: Human Telomerase Reverse Transcriptase
TA: Telomerase Activity
TRAP: Telomeric Repeat Amplification Protocol
GC: Granulosa Cells
GTL: Granulosa Telomere Length
qPCR: Quantitative Polymerase Chain Reaction
